# Feasibility of Measuring GABA Levels in the Upper Brainstem in Healthy Volunteers Using Edited MRS

**DOI:** 10.3389/fpsyt.2020.00813

**Published:** 2020-08-14

**Authors:** Yulu Song, Tao Gong, Richard A. E. Edden, Guangbin Wang

**Affiliations:** ^1^ Department of Imaging and Nuclear Medicine, Shandong Medical Imaging Research Institute, Cheeloo College of Medicine, Shandong University, Jinn, China; ^2^ Russell H. Morgan Department of Radiology and Radiological Science, The Johns Hopkins University School of Medicine, Baltimore, MD, United States; ^3^ FM Kirby Center for Functional Brain Imaging, Kennedy Krieger Institute, Baltimore, MD, United States

**Keywords:** GABA, MRI, MEGA-PRESS, brainstem, spectroscopy

## Abstract

**Purpose:**

To assess the feasibility of small-voxel MEGA-PRESS in detecting gamma-aminobutyric acid (GABA) levels of the upper brainstem in healthy volunteers.

**Materials and Methods:**

Forty-two healthy volunteers, aged between 20 and 76 years were enrolled in this study, and underwent a 3.0T MRI scan using an eight-channel phased-array head coil. The MEGA-PRESS sequence was used to edit GABA signal from a 10x25x30 mm^3^ voxel in the upper brain stem. The detected signal includes contributions from macromolecules (MM) and homocarnosine and is therefore referred to as GABA+. All the data were processed using Gannet.

**Results:**

Thirty-four cases were successful in measuring GABA in the upper brainstem and 8 cases failed (based on poor modeling of spectra). The GABA+ levels were 2.66 ± 0.75 i.u. in the upper brainstem of healthy volunteers, ranging from 1.50 to 4.40 i.u. The normalized fitting residual (FitErr in Gannet) was 12.1 ± 2.8%, ranging from 7.4% to 19.1%; it was below 15.5% in 30 cases (71%).

**Conclusions:**

It is possible to measure GABA levels in the upper brainstem using MEGA-PRESS with a relatively small ROI, with a moderate between-subject variance of under 30%.

## Introduction

γ-aminobutyric acid (GABA) is the major inhibitory neurotransmitter in the developmentally mature mammalian central nervous system. GABA acts at inhibitory synapses in the brain by binding to specific transmembrane receptors in both pre- and postsynaptic neuronal processes. ^1^H magnetic resonance spectroscopy (MRS) is a noninvasive technique that can be used to measure neurotransmitter levels in vivo (1). However, GABA is difficult to detect due to its low concentration and the presence of overlapping signals from other compounds such as creatine and N-acetyl aspartate (NAA) (2). Mescher-Garwood point-resolved spectroscopy (MEGA-PRESS) is able to estimate GABA levels reliably using an editing technique based on refocusing J-couplings (3). To date, MEGA-PRESS has been measured both in the healthy brain (4–8) and in various neurodegenerative disorders, such as Alzheimer’s disease (AD) (9, 10), multiple sclerosis (MS) (11, 12), as well as psychiatric diseases (13–16).

In the human brain, the brainstem includes the midbrain and the pons and medulla oblongata of the hindbrain. The midbrain is the recipient of projections from cortex, limbic structures, and striatum, and exerts essential modulating influences on those descending and ascending projections (17, 18). The upper brainstem (19) is under-studied by MRS, even though it contains many integrative nuclei that mediate physiological functions disrupted in neurological disease (18). Numerous studies have been performed on the pathological brainstem involvement in patients with neurodegenerative disorders (20–22). One QSM study showed that the pathological changes in the midbrain (elevated iron) have a direct relevance to the development of Parkinson’s Disease (PD) (23). Zarow et al. found that for both PD and AD the greatest neuronal loss was found in the pons (Locus coeruleus, LC), and demonstrated that neural loss in the pons and midbrain was correlated with the duration of these neurodegenerative disorders (24). Some found that the sites in the upper brainstem that are damaged are likely to contribute to the physiological deficits emerging in PD and obstructive sleep apnea (OSA) (18, 25). One study of GABA+ at 7 T in PD showed a correlation between GABA levels in the pons and the putamen (22). To our knowledge, no study has yet sought to measure GABA+ levels in the upper brainstem at 3T using MEGA-PRESS, likely because the small volume in upper brainstem makes voxel sizes (~27ml) used in most previous studies impossible (6, 26–29). The objectives of this preliminary study were to measure the GABA+ levels of the upper brainstem in a small voxel (10 x 25 x 30 mm^3^) with MEGA-PRESS in healthy volunteers.

## Methods

### Participants

Between April 2018 and April 2019, 42 healthy volunteers (55% males and 45% females aged 20–76 years) were recruited to be scanned by 3T MRI. Written informed consent was obtained from each participant. The study was approved by the local institutional ethical review board. For all participants, exclusion criteria included contraindications for MRI and a history of alcohol or substance misuse.

### MRS Acquisition

All MRI and MRS experiments were carried out using a 3T scanner (Achieva TX, Philips, Best, Netherlands) equipped with an eight-channel phased-array head coil.

#### Magnetic Resonance Spectroscopy

**Figure 1:**
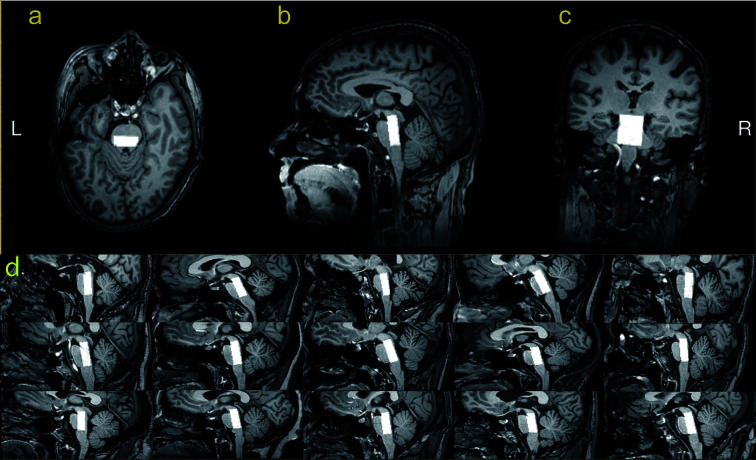
T1-weighted TFE images show the ROI’s position in the upper brainstem in a healthy volunteer. The white box represents the location of the ROI (10mm*25mm*30mm) in the axial **(A)**, sagittal **(B)**, and coronal **(C)** planes. Sagittal placement in 15 subjects illustrates the variable geometry and adjusted placement **(D)**.

The MEGA-PRESS sequence generates two subspectra with the editing pulse ON in one and OFF in the other for GABA detection. The edited spectrum, which reveals the GABA+ signal, is obtained by subtracting one subspectrum from the other (30). MEGA-PRESS spectra were acquired from a 10 x 25 x 30 mm^3^ voxel in the upper brainstem. Based on axial images, the voxel was centered left-right and in the midbrain, with the posterior face aligned to the posterior face of the midbrain. Based on sagittal images, the voxel was positioned cranially/caudally so as to maximize inclusion of midbrain and pontine tissue, and minimizing the inclusion of cerebrospinal fluid, as shown in **Figure 1**. **Figure 1D** illustrates the typical voxel placement across 15 subjects.

Sequence parameters were as follows: repetition time (TR) = 2 s; echo time (TE) = 68 ms; 320 averages; acquisition bandwidth = 2,000 Hz; Total acquisition time 10 min 56 sec; MOIST water suppression (31) and Philips pencil-beam (PB-auto) shimming (32) were used. Because the signals detected at 3.02 ppm using these experimental parameters is also expected to contain contributions from both macromolecules (MM) and homocarnosine, in this study the signal is labeled GABA+ rather than GABA (22).

#### Structural Magnetic Resonance

A whole-brain structural 3-dimensional MPRAGE (magnetization-prepared rapid-acquisition gradient echo) scan was acquired with 1 x 1 x 1 mm^3^ isotropic resolution (TR = 8.2 ms; TE = 3.7 ms; flip angle = 8°, matrix = 256 × 256; field of view = 24 × 24 cm^2^).

#### Magnetic Resonance Spectroscopy Data Analysis

MRS data were analyzed using the Gannet 3.1 software toolkit, a GABA-MRS analysis tool that is coded within MATLAB (The MathWorks, Inc., Natick, MA, USA) using the Optimization and Statistics toolboxes and is distributed as open-source software (33, 34). The analysis consisted of the following steps: (1) frequency-and-phase alignment of FIDs with robust spectral correction (35); (2) averaging and subtraction of aligned spectra to produce GABA+ spectra; (3) fitting a Gaussian to the 3-ppm GABA+ peak to quantify GABA+ based on the area under the curve. GABA+ levels were quantified in institutional units (i.u.) relative to the unsuppressed water signal. The version of Gannet used for this analysis has been archived on OSF at: https://osf.io/q92mb/.

The primary data quality metric generated by Matlab is fitting error (FitErr,%), calculated as the ratio of the standard deviation of the fitting residual to the GABA+ signal amplitude. A data quality cut-off of 15% (11, 27, 36) has been applied previously for brain applications with good SNR. In this study, we used a cut-off of 20% (37) to reject spectra of poor quality, using a less stringent criterion to reflect the lower SNR of the smaller-VOI (voxel of interest) brainstem acquisition. Using a 20% cut-off allows us to retain a larger number of datasets in the analysis, at the cost of increasing the variability of data to include poorer-quality data. The choice of an appropriate cut-off for future *in vivo* studies is a trade-off between variance and sample size (which both impact statistical power) and possible inclusion bias, e.g. only including data from more compliant subjects.

Metrics of GABA+ SNR and creatine signal linewidth are also calculated by Gannet and reported.

### Statistical Analysis

Due to the large age-range of subjects, a secondary statistical analysis was performed to investigate whether the midbrain GABA+ level was correlated with age. The average GABA+ concentrations for the male and female participants were compared with a two-tailed unpaired *t*-test, using the concentrations derived with Gannet 3.1. Statistical analysis was performed in the Statistical Package for Social Sciences (SPSS version 20.0; IBM Corp., Armonk, NY). To evaluate age-related differences in GABA+ of upper brainstem, the Pearson correlation coefficient was calculated and a Fisher R-to-z transforms to assess significance with a *p*-threshold of 0.05.

## Results

MRI scans were obtained on 42 healthy volunteers. **Table 1** shows the demographics of these participants. The volunteer group is with mean age: 45.7 ± 14.7 years (19 females, 45.2%; 23 males, 54.8%).

**Table 1:** Demographics of all subjects.

Demographics of the participants.	
	HC
Total number of subjects	42
Age in years	45.7 ± 14.7
No.of female (%)	19 (45.2%)
No.of male (%)	23 (54.8%)

**Figure 2:**
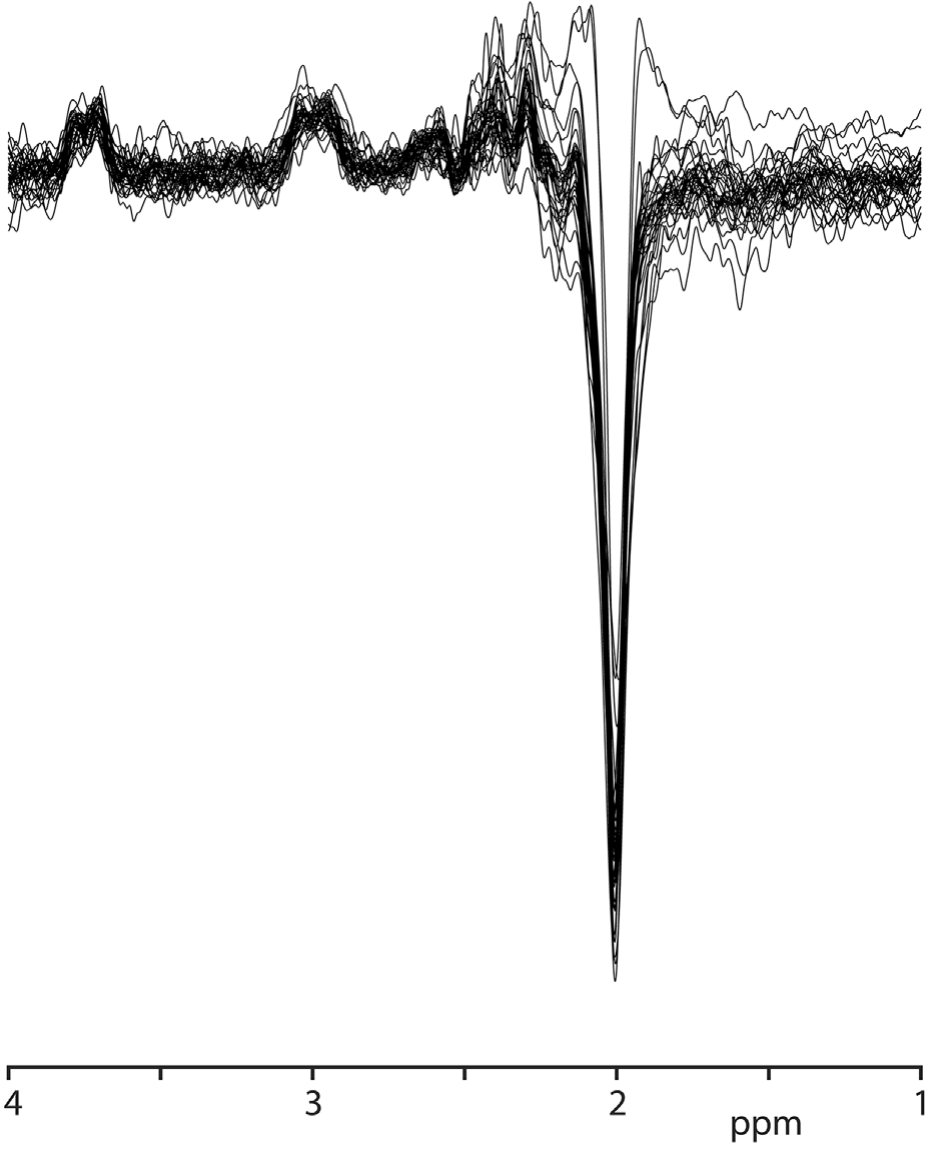
In vivo GABA-edited spectra from the upper brainstem from 34 subjects (overlaid).

**Figure 3:**
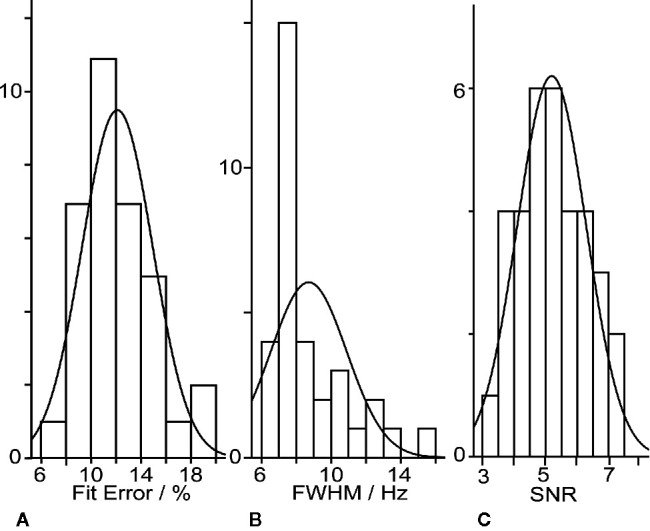
Histograms of the distributions of fitting errors **(A)**, linewidth **(B)**, SNR **(C)**.

GABA+ levels were 2.66 ± 0.75 i.u. in the healthy volunteers, ranging from 1.50 to 4.40 i.u (as shown in **Table 2**). The mean GABA FitErr for upper brainstem voxel of volunteers was 12.1 ± 2.8% (spectra with fitting error below 20% are shown in **Figure 2**). The median FitErr was 11.7%, and as can be seen in **Figure 3A**, the distribution of fitting errors is only moderately skewed (skewness = 0.772). 8 cases have a fitting error below 10%, 20 between 10 and 15%, 6 cases 15-20% and 8 cases over 20%, which were omitted from the analysis.

**Table 2:** Subject demographics and MRS results.

	number	gender	Age	[GABA+]/i.u.	GABA+/Cr	FitErr (%)	GABA+ SNR	Cr.FWHM (linewidth)
1	MH-90*	F	55	61.89	2.024	2.4	47.3397	11.5112
2	MH-34	M	76	3.66	0.275	7.4	6.7847	6.6656
3	MH-116	M	57	4.397	0.228	8.4	3.8804	7.4313
4	MH-100	F	24	3.69	0.211	8.6	6.83	11.4299
5	MH-47	M	66	2.278	0.159	8.9	5.4383	7.3013
6	MH-95	M	48	2.892	0.148	9.3	6.5632	8.3427
7	MH-99	F	24	3.539	0.219	9.4	5.6473	6.8646
8	MH-107	M	56	2.623	0.124	9.7	3.8066	8.3243
9	MH-114	M	59	4.055	0.209	9.7	5.8652	7.349
10	MH-103	F	24	2.58	0.134	10	7.2623	9.7712
11	MH-104	M	56	2.731	0.156	10.2	4.5534	7.4724
12	MH-66	F	27	1.674	0.09	10.6	6.4	7.9068
13	MH-69	M	29	2.38	0.11	10.7	5.189	13.21
14	MH-27	F	50	3.375	0.092	10.7	7.0929	15.2452
15	MH-94	M	60	2.796	0.148	10.9	4.8486	7.187
16	MH-106	F	52	3.455	0.169	11.1	4.6792	9.2192
17	MH-37	F	36	2.586	0.203	11.4	5.7647	6.2624
18	MH-97	F	24	2.294	0.088	11.7	4.6513	10.9155
19	MH-92	M	56	2.537	0.143	11.7	4.5603	7.5527
20	MH-23	F	25	3.271	0.188	11.9	5.1726	7.7503
21	MH-101	M	24	3.374	0.299	12	5.433	6.4064
22	MH-110	F	58	3.318	0.179	12.4	6.4166	7.049
23	MH-61	M	56	2.209	0.119	12.7	4.0212	7.703
24	MH-73	F	24	1.52	0.01	12.8	5.0426	8.74
25	MH-115	F	50	2.937	0.112	13.2	5.6654	10.2316
26	MH-78	F	24	1.971	0.086	13.6	5.0123	10.00081
27	MH-117	F	59	2.329	0.122	13.8	6.2853	7.601
28	MH-112	M	36	2.209	0.114	14	3.7992	8.0255
29	MH-113	M	49	2.608	0.143	14.2	6.0098	7.7374
30	MH-108	M	52	1.678	0.065	15.1	4.1782	12.8079
31	MH-86	F	47	1.712	0.068	15.4	3.7966	12.3237
32	MH-109	M	60	2.131	0.101	15.6	4.0376	8.3851
33	MH-96	F	24	1.503	0.085	17.5	3.2026	7.5706
34	MH-53	M	61	2	0.106	18.1	4.6171	7.6339
35	MH-111	M	38	2.012	0.102	19.1	4.085	7.8425
36	MH-105*	M	57	2.042	0.118	20.1	3.3165	12.0987
37	MH-93*	M	53	1.815	0.078	23.6	3.9638	9.027
38	MH-89*	M	44	1.11	0.006	25	1.4629	7.8561
39	MH-91*	F	61	1.71	0.009	26	2.3535	6.5642
40	MH-87*	M	55	1.001	0.004	33	1.2958	7.0623
41	MH-98*	M	40	2.12	0.111	33	1.2958	7.0623
42	MH-88*	F	45	2.11	0.077	45.4	1.1705	6.8163

*Denotes low-quality data with FitErr >20%.

The SNR is highly dependent on the experimental conditions and is modified by such factors as the field strength, voxel size, total acquisition time, TR, TE, J-coupling modulation, coil loading, and the number of nuclei in the sample. In this study the mean SNR for upper brainstem voxel of volunteers was 5.19 ± 1.09 (as seen in **Figure 3**). The median SNR was 5.11. The distribution of SNR is only moderately skewed (skewness = 0.164 > 0). The linewidth is determined by a combination of the inherent T2 of the spin system under consideration and the loss of phase coherence in the sample volume from B_0_ inhomogeneity caused by local differences in magnetic susceptibility. In our study the mean Cr linewidth was 8.7 ± 2.2 Hz. The median linewidth was 7.8 Hz (as seen in **Figure 3**). The distribution of linewidths is only moderately skewed (skewness = 1.5 > 0).

No significant correlations (p=0.215 > 0.05) with age were observed. There is no significant gender-related differences in the GABA+ levels in upper midbrain in the healthy volunteers (p=0.735>0.05).

## Discussion

This study demonstrates the feasibility of GABA-edited MEGA-PRESS in the upper brainstem in a moderately sized cohort of healthy volunteers and serves as an important foundation for future patient studies in movement disorders.

The brainstem is a midline structure formed by the midbrain, pons, and medulla containing a number of critical substructures (38). The major dopaminergic (DA) neuronal population of the mammalian brain is located in the ventral midbrain (VM) (39). The role of the brainstem as a route for all major efferent and afferent pathways to the periphery relies heavily on GABAergic connectivity (17). Brainstem pathology is implicated in many neurodegenerative disorders, including PD (20, 21). Postmortem evidence in PD indicates that pathological changes in the pons and medulla precede those in the substantia nigra, a key area of neuronal loss (22). It is therefore desirable to develop an MRS measure of GABA in the brainstem.

Edited MRS of GABA is made challenging by the relatively low concentration of GABA, signal overlap, and large typical voxel sizes. In order to measure GABA levels in the brainstem, a small-region of interest (ROI) MEGA-PRESS protocol has been developed. In spite of the relatively unfavorable anatomy, the linewidth achieved (median 7.8 Hz) is comparable to that seen in parietal lobe of the Big GABA multi-site study (mean 7.7 Hz (31)). This narrow linewidth is aided by the small voxel size (10 x 25 x 30 mm^3^, compared to typical volumes of 30 x 30 x 30 mm^3^ in the brain (31)). The GABA SNR is substantially lower than the SNR in that study (5.1 vs. 25), which reduction is largely explained by the 72% reduction in acquisition volume. The remaining reduction in SNR is likely attributable to the greater distance between the midbrain and the receive coil elements.

While the brainstem is under-studied with MRS compared to many cortical regions, there is some literature applying conventional (unedited) MRS there. For example, one MRS study at 1.5T showed higher N-acetylaspartate (NAA)/creatine (Cr) ratios in a 7.5 x 7.5 x 10 mm^3^ voxel in bilateral rostral dorsal pons in patients with episodic migraine (EM) than those in patients with chronic migraine (CM) and normal controls (40). This study initiated the MRS scan if the water linewidth reported by the prescan process was less than 6 Hz. The ability of linear-combination modeling of conventional spectra and to resolve GABA signals without editing at 3T (and 7T) remains the subject of discussion. Macky et al. conducted conventional (unedited) PRESS-localized MRS at 3T in the midbrain (15 x 15 x 15 mm^3^ voxel) and reported no differences in the ratio of GABA:creatine in patients with OSA compared with controls (18). Uzay et al. performed unedited MRS at 7T in the midbrain (30 x 10 x 15 mm^3^ voxel), and showed the GABA+ level was increased in the pons in 11 patients with PD. In controls, the GABA+ level was 1.0 ± 0.2 mmol/g, with substantially larger relative variance than our study. One prior MEGA-PRESS pilot study showed GABA+ levels were significantly lower in the brainstems(25×18×30) of 12 possible Sleep Bruxism (SB) patients compared with 12 controls (41). This study conducted higher-order shimming to achieve linewidths of <25 Hz.

A number of studies have suggested that there is an age-related decrease of GABA+ levels in frontal and parietal regions in healthy control subjects (4, 42–44). While this effect is largely driven by bulk tissue changes, GABA+ levels still correlate with cognitive function (42). No MRS studies have investigated age-related changes in GABA+ concentrations in the upper brainstem of HC. The current study found no significant relationship between GABA+ levels and age. It is not currently possible to determine whether this negative result is due to reduced sample size, increased measurement variance or reduced neurodegenerative aging of the brainstem. Previous studies examining age-related volumetric decline in the brainstem have found no age effects for the volume of the whole brainstem, metencephalon or medulla, with only the midbrain showing a trend for age-related shrinkage (45, 46). In our study, the ROI mainly consists of midbrain and pons. Besides, a number of studies have suggested that there is a gender-related difference of GABA+ levels in dorso-lateral prefrontal cortex (4, 47). However, one edited-MRS study (48) showed that there is no gender differences were detected in anterior cingulate cortex, which was consistent with our study. The current study found no significant relationship between GABA+ levels and gender.

An eight-channel head coil was employed in our study. It is likely that higher-order, e.g. 32-channel, coils and other hardware innovations such as digital receiver chain would deliver higher SNR data that reported here. Measures of gray and white matter volume, white matter connectivity (fractional anisotropy and diffusivity measures) and functional connectivity in resting state networks are improved with 32- rather than 8-channel receive hardware (49). One MRS study showed that 32-channel would be better than 8 or 16-channel in SNR variations due to different hardware, pulse sequences, and post-processing between imaging and spectral scans (50). However, in our results, the SNR (mean: 5.19 ± 1.09; median: 5.11) is acceptable. We are confident that this demonstration of feasibility on an 8-channel head coil is applicable to other scanners with 32-channel hardware.

This study has several limitations. Firstly, the MEGA-PRESS sequence parameters give a GABA+ signal contaminated with MM and homocarnosine signals. Editing-based methods have been proposed to suppress the MM contribution (51), but the degree of MM suppression (and the polarity of the MM residual signal) is highly dependent on changes in the scanner frequency associated with motion and gradient heating (6). Secondly, the small size of the brainstem requires a small voxel. This results in a GABA+ measurement that has substantially lower SNR than is commonly achieved for brain measurements using a larger voxel. However, even though the voxel size is small for GABA MRS, it is large relative to the complex internal anatomy of the brainstem and includes a number of distinct structures without differentiation. This is a limitation inherent to MRS of low-concentration metabolites. We also recognize that the lack of a gold standard for validation is a general concern for MRS measurements. Phantom validation of MRS protocols can be performed (and will indicate good linearity of measurements), but the challenges of MRS in the brainstem mostly center around subject and physiological motion, and local magnetic inhomogeneity, which phantom measurements do not address.

In conclusion, we have demonstrated that it is feasible to measure GABA+ levels in the upper brainstem using MEGA-PRESS. Understanding alterations in upper brainstem neurotransmitters will provide insight into pathological dysfunction in neurodegenerative disorders.

## Data Availability Statement

All datasets generated for this study are included in the article/supplementary material.

## Ethics Statement

The studies involving human participants were reviewed and approved by the Ethics Committee of Shandong Medical Imaging Research Institute. The patients/participants provided their written informed consent to participate in this study. Written informed consent was obtained from each participant for the publication of any potentially identifiable images or data included in this article.

## Author Contributions

YS and TG contributed conception and design of the study. YS and TG organized the database. YS performed the statistical analysis. YS wrote the first draft of the manuscript. RE and GW revised the manuscript. All authors contributed to the article and approved the submitted version. The corresponding author GW takes primary responsibility for communication with the journal and editorial office during the submission process, throughout peer review, and during publication. The corresponding author GW is also responsible for ensuring that the submission adheres to all journal requirements including, but not exclusive to, details of authorship, study ethics and ethics approval, clinical trial registration documents and conflict of interest declaration. The corresponding author GW should also be available post-publication to respond to any queries or critiques.

## Funding

This study applies tools developed under NIH grants R01 EB016089 and P41 EB015909; RAEE also receives salary support from these grants. This project was also funded by National Natural Science Foundation of China(81671668;81371534), Major research project of Shandong province(2016ZDJS07A16). Wang G also receives salary support from these grants.

## Conflict of Interest

The authors declare that the research was conducted in the absence of any commercial or financial relationships that could be construed as a potential conflict of interest.
